# Mixed Solvents Assisted Post‐Treatment Enables High‐Efficiency Single‐Junction Perovskite and 4T Perovskite/CIGS Tandem Solar Cells

**DOI:** 10.1002/advs.202201768

**Published:** 2022-06-08

**Authors:** Liting Tang, Xiaomin Wang, Xinxing Liu, Junjun Zhang, Shaoying Wang, Yuqi Zhao, Junbo Gong, Jianmin Li, Xudong Xiao

**Affiliations:** ^1^ Key Laboratory of Artificial Micro‐ and Nano‐Structures of Ministry of Education and School of Physics and Technology Wuhan University Wuhan 430072 China; ^2^ Center for Biomedical Optics and Photonics (CBOP) & College of Physics and Optoelectronics Engineering Key Laboratory of Optoelectronic Devices and Systems Shenzhen University Shenzhen 518060 P. R. China

**Keywords:** copper indium gallium selenide, interface passivate, mixed solvents, perovskite, tandem solar cells

## Abstract

The interface between the perovskite layer and the hole transport layer (HTL) plays a vital role in hole extraction and electron blocking in perovskite solar cells (PSCs), and it is particularly susceptible to harmful defects. Surface passivation is an effective strategy for addressing the above concerns. However, because of its strong polarity, isopropyl alcohol (IPA) is used as a solvent in all of the surface treatment materials reported thus far, and it frequently damages the surface of perovskite. In this paper, a method is proposed for dissolving the passivation materials, for example, guanidine bromide (GABr), in mixed solvents (1:1) of IPA and toluene (TL), which can efficiently passivate interface and grain boundary defects by minimizing the IPA solubility of the perovskite surface. As a result, all the performance parameters Voc, Jsc, and FF are improved, and the power conversion efficiency (PCE) increased from 20.1 to 22.7%. Moreover, combining the PSCs with GABr post‐treatment in mixed solvents with copper indium gallium selenide (CIGS) solar cells, a 4‐terminal (4T) perovskite/CIGS tandem device is realized and a PCE of 25.5% is achieved. The mixed solvent passivation strategy demonstrated here, hopefully, will open new avenues for improving PSCs’ efficiency and stability.

## Graphical abstract

22.7%‐Efficient perovskite solar cell and 25.5%‐efficient perovskite/CIGS tandem solar cell are achieved by mixed solvents assisted GABr post‐treatment strategy which effectively improves the quality of perovskite film, passivates defect and thus minimizes non‐radiative recombination.

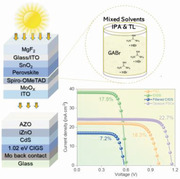

## 1 Introduction

Perovskite has stimulated numerous interest for researchers in the last 10 years due to its excellent photoelectric properties including adjustable band gaps, large visible light absorption coefficient, and high power conversion efficiency (PCE) of perovskite solar cells (PSCs).^[^ ^1^ ^]^ Since its inception in 2009, researchers have made continuous improvements in composition engineering, perovskite‐transport layer interface engineering, and solvent engineering.^[^ ^2^, ^3^, ^4^ ^]^ Currently, the single‐junction PSCs have been reported to achieve a recorded PCE of 25.8%, making perovskite solar cells a disruptive type of photovoltaic technology.^[^ ^5^, ^6^ ^]^ Because of its bandgap tunability from 1.17 eV to 3.10 eV, perovskite is also an ideal choice as both wide‐bandgap top and narrow‐bandgap bottom sub‐cells in tandem solar cells (TSCs).^[^ ^7^, ^8^, ^9^ ^]^ Combining a wide‐bandgap perovskite top cell and a narrow‐bandgap bottom cell, either based on mixed tin (Sn)‐lead (Pb) perovskite or based on a dissimilar material such as silicon (Si) or copper indium gallium selenide (CIGS), to form tandems can offer an extraordinary opportunity to go beyond the Shockley‐Queisser (S‐Q) efficiency limit of single‐junction solar cells.^[^ ^10^, ^11^, ^12^ ^]^

Since both perovskite and CIGS solar cells use thin‐film technology, perovskite/CIGS tandem solar cells can be manufactured on thin, lightweight, and flexible substrates in a cost‐effective manner, and therefore can offer enormous potential to wide application opportunities, including building‐integrated photovoltaics (BIPV) and mobile power devices.^[^ ^12^, ^13^, ^14^ ^]^ Compared to the two‐terminal (2T) architecture, the mechanically‐stacked four‐terminal (4T) device has reduced constraints from fabrication process compatibility and current matching and provides much more flexibility for each sub‐cell structure design.^[^ ^15^ ^]^ Using a 12.7% efficiency semi‐transparent n‐i‐p PSC based on a silver nanowire contact, Bailie et al. were the first to demonstrate the feasibility of perovskite/CIGS tandem with an efficiency of 18.6%.^[^ ^14^ ^]^ The recently reported world record efficiency for a 4T perovskite/CIGS tandem is 25.9%, contributed by a p‐i‐n structured PSC, and has surpassed both the single‐junction CIGS and PSC record efficiency.^[^ ^16^ ^]^ Further development should focus on continuous improvement of the semitransparent top perovskite devices and the selection of bottom sub‐cells.

Usually, charge carriers may be trapped by deep‐level defects at the surface and interface of perovskite films, resulting in charge accumulation, recombination loss, and hysteresis, and lowering device performance.^[^ ^17^, ^18^ ^]^ Perovskite films, normally formed through solution processing and high‐temperature crystallization, inevitably own a large number of detrimental ion vacancies and dangling bonds on the perovskite surface, leading to serious recombination losses in the film.^[^ ^19^ ^]^ With a faster recombination rate than defects inside grains or at grain boundaries, defects on the perovskite surface (top and bottom) dominate the charge carrier lifetime.^[^ ^20^ ^]^ Furthermore, the Spiro‐OMeTAD is commonly utilized as the hole transport layer (HTL) in today's most efficient PSCs systems, which necessitates the addition of Lithium Bis(trifluoromethanesulfonyl)imide salt (Li‐TFSI) and 4‐tert‐butylpyridine (tBP) to boost carrier mobility. However, due to the moisture absorption of Li‐TFSI and the corrosive impact of tBP on the perovskite layer, the decomposition of the perovskite film will be accelerated.^[^ ^21^ ^]^ Therefore, optimizing the interface between the perovskite layer and the HTL is critical for PSCs performance. Recently, there have been some reports on the treatment of the interface between perovskite and HTL. Organic halides^[^ ^17^, ^22^, ^23^, ^24^ ^]^ like phenethylammonium iodide (PEAI), ammonium iodide butyrate (BAI), octylammonium iodide (OAI), and GABr, for example, can passivate defects at the perovskite interface, improving the open‐circuit voltage (Voc) and fill factor (FF), and hence the device performance of PSCs.^[^ ^25^ ^]^ During the treatment process, one of the most important questions to consider is the solvent, which plays a key role in controlling morphology, broadening the process window, and influencing the performance of PSCs. At present, solvent selection of perovskite precursor solution, Spiro‐OMeTAD solution, and anti‐solvent have been studied and reported.^[^ ^26^, ^27^, ^28^, ^29^, ^30^ ^]^ With the continuous improvement of PSCs efficiency, it is also worthwhile to investigate the solvent selection of surface passivation materials.^[^ ^31^ ^]^ Isopropyl alcohol (IPA),^[^ ^22^, ^32^, ^33^, ^34^ ^]^ acts as a popular solvent, has been used in most studies of PSCs surface treatment. While it is excellent in dissolving passivated materials, unfortunately it can also dissolve the perovskite layer, particularly formamidine iodide (FAI)‐based films.^[^ ^35^ ^]^ As a result, poor reproducibility, device instability, and other issues are prevalent when using it to passivate perovskite film surfaces.^[^ ^35^ ^]^

In this work, to overcome the above problems, we propose a new strategy for post‐treating perovskite films that involves dissolving GABr in mixed solvents (denoted by MS‐GABr). As well known, ethyl acetate (EA), chlorobenzene (CB), and toluene (TL) are less polar solvent than IPA.^[^ ^26^ ^]^ We compared the mixing of IPA with EA, CB, and TL to dissolve GABr, and further optimized the concentration of GABr and the mixing ratio of the two solvents. Our experiment discovered that when IPA and TL were mixed at a concentration of 2 mg mL^−1^ and dissolved at a ratio of 1:1, the mixed solvents assisted treatment may not only minimize perovskite surface solubility, but also efficiently passivate interface and grain boundary defects, reduce non‐radiative recombination of the film surface, and avoid poor heterogeneous carrier transmission. As a result, the device's performance parameters have been improved: the open‐circuit voltage (Voc) rises by 90 mV, the short‐circuit current density (Jsc) increases from 23.6 mA cm^–2^ to 24.2 mA cm^–2^, and the fill factor (FF) improves from 77.9% to 80.6%. In the end, the efficiency of the PSCs can be increased from 20.1% to 22.7%. We have further adopted this strategy in semi‐transparent perovskite solar cells and perovskite/CIGS tandem solar cells preparation. Consequently, the semi‐transparent perovskite solar cells fabricated with MS‐GABr solution treatment and a transparent electrode made of sputtered tin‐doped indium oxide (ITO) on MoO_X_ buffer, have achieved an efficiency of 18.3%. Combined with a 17.5% efficiency CIGS device, a 4T perovskite/CIGS tandem solar cell of 25.5% was realized.

## 2 Results and Discussion

As we know, compared with IPA, GABr has poor solubility in EA, CB, TL, and other less polar solvents. As shown in Figure S1a–d, Supporting Information, 2 mg mL^−1^ GABr was dissolved in IPA, EA, CB, and TL, respectively. It is obvious that the GABr can only completely dissolved in IPA, but not in EA, CB, and TL. Moreover, the TL has the lowest solubility and the solution becomes cloudy. However, unexpectedly, as shown in Figure S1e–g, Supporting Information, GABr can be completely dissolved at a concentration of 2 mg mL^−1^ in the 1:1 mixed solvent of IPA with EA, CB, and TL (denoted by G‐I/EA, G‐I/CB, and G‐I/TL solution respectively). Based on this discovery, in this work, three types of MS‐GABr are introduced in the post‐treatment process. For comparison, the samples without post‐treatment and treated with GABr solution in a single IPA (G‐I) solvent are also listed.

**Scheme 1:**
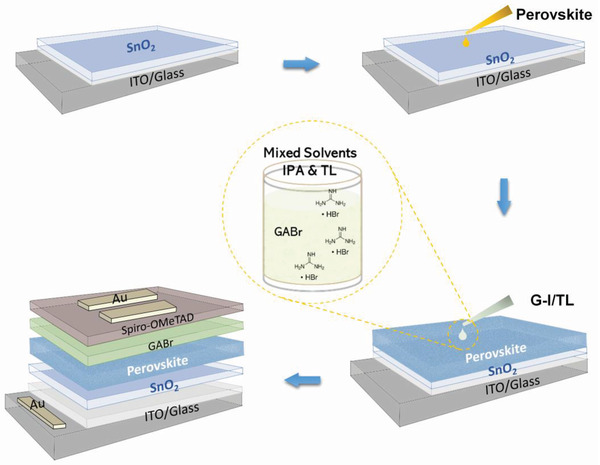
Schematic fabrication process of a planar perovskite solar cell with GABr‐IPA & TL (denoted by G‐I/TL) solution treatment.

**Figure 1:**
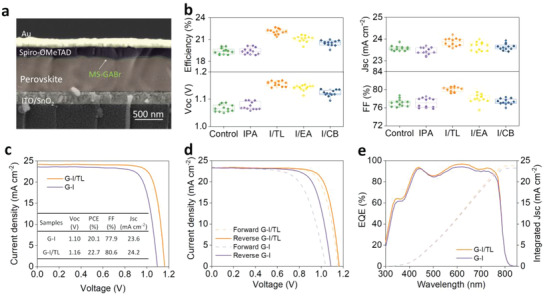
a) Cross‐sectional SEM image of planar perovskite solar cells treated by G‐I/TL solution. b) Performance parameters of solar cells with and without GABr treatment in different solvents. c) J‐V curves of the champion devices based on G‐I and G‐I/TL treatments. d) Forward and reverse scan of G‐I and G‐I/TL‐based devices. e) EQE spectra of the champion devices. The integrated current densities are 23.2 and 23.8 mA cm^–2^, respectively.

**Scheme** **1** shows the typical structure of perovskite solar cells and a schematic of the post‐treatment strategy. We first mixed the IPA with EA, CB, and TL (1:1) respectively to dissolve GABr (2 mg mL^−1^), and then spin‐coated it on the surface of the perovskite layer. Finally, the planar perovskite solar cells were constructed by spin‐coated Spiro‐OMeTAD HTL and thermally evaporated Au layers. For more details, please refer to Section 4. **Figure** **1**a shows the cross‐sectional SEM image of the G‐I/TL treated device with ITO/SnO_2_/perovskite/Spiro‐OMeTAD/Au structure. Generally, the performance of solar cells is the foremost standard for the assessment of the mixture solvent treatment strategy. Hence, we first compare the performance parameters of the devices based on perovskite films treated with the above four types of solution and the one without any treatment (denoted by the control group). The statistics of performance parameters for a total of 60 devices are shown in Figure 1b. It is interesting and encouraging to see that the devices treated by solution with mixture solvent show better performance than those treated by solution with a single solvent and without any treatment. Moreover, no obvious enhancement is found for devices treated with a single solvent (IPA) as compared to the one without any treatment. Among these solar cells treated by solutions with mixture solvent, the G‐I/TL treated devices show the best performance. By systematically optimizing the concentration of GABr (Figure S2, Supporting Information), the ratio of IPA: TL (Figure S3, Supporting Information), and annealing parameters (Figure S4, Supporting Information), the best device with a PCE of 22.7% have been obtained with 2 mg mL^−1^ GABr dissolved in 1:1 ratio of IPA: TL mixture solvents and without extra annealing process. To reveal the role of mixed solvents, we further compared G‐I with G‐I/TL treated devices. By careful comparison of the parameters between the two best devices fabricated with G‐I and G‐I/TL solution, as shown in Figure 1d and Figure S5, Supporting Information, it is found that all the parameters of devices treated by solution with mixture solvent are higher. The Jsc is increased from 23.6 mA cm^–2^ to 24.2 mA cm^–2^, the Voc from 1.10 V to 1.16 V, and the FF from 77.9% to 80.6%, respectively. These results reveal that the better performance of MS‐GABr treated devices is a result of the synergistic effect of mixed solvents during the treatment process.

To further explore the differences between the two types of cells, the hysteresis and EQE measurements were made and the results are displayed in Figure 1e,f. Ion migration, high trap density, and unbalanced charge transport are currently considered to be the main causes of hysteresis.^[^ ^36^ ^]^ The hysteresis index (Hysteresis) of the devices is calculated based on Equation (1).^[^ ^37^ ^]^

(1) $$\mathrm{Hysteresis}=\left(\mathrm{PC}E_{\mathrm{reverse}}-\mathrm{PC}E_{\mathrm{forward}}\right)/\mathrm{PC}E_{\mathrm{reverse}}\times 100\%$$

In this study, the forward and reverse scan J‐V curves of all types of devices are shown in Figure S6a–e, Supporting Information, along with the calculated Hysteresis in Table S1, Supporting Information. Among them, the G‐I/TL treated device has the lowest hysteresis index of 3.8%. In terms of EQE results, the G‐I/TL treated device exhibits an evident rise across the whole spectral range, as shown in Figure 1f and Figure S7, Supporting Information, indicating that the G‐I/TL solution treatment effectively promoted charge extraction and corresponds well with the I‐V test result. At this point, it is reasonable to conclude that devices treated with MS‐GABr have better‐quality perovskite layers. The specifics will be covered in the following section.

**Figure 2:**
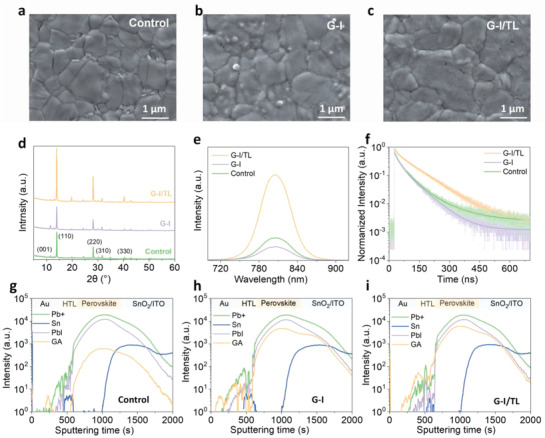
Structural, optical, and constitutive properties of perovskite layers without treatment, with G‐I/TL, and G‐I solution treatment, respectively. a–c) Top‐view SEM images. d) X‐ray diffraction (XRD) patterns. e,f) Steady‐state PL and TRPL spectra. g–i) The ToF‐SIMS results of the corresponding devices.

As mentioned above, the devices treated with solution of mixture solvents show better performance. To reveal the mechanism behind this improvement, the structural and optical properties of these perovskite layers with different treatments were first studied. In general, the quality of perovskite films can be defined by some significant characteristics, such as grain size, crystallinity, and surface coverage, which together affect the performance of perovskite solar cells.^[^ ^38^ ^]^ **Figure** **2**a–c shows the top‐view SEM images of three types of perovskite layers, including the films without treatment, with G‐I/TL and G‐I solution treatment. Surprisingly, the films treated with MS‐GABr possess the largest grain size and the most uniform surface, presumably reducing defects at grain boundaries and interfaces, which is a desirable feature for planar thin‐film solar cells with good performance. Furthermore, as shown in Figure S8, Supporting Information, the perovskite surface is breached by the pure IPA solvent, whereas the IPA/TL mixture solvent and pure TL solvent show very little dissolution of the perovskite samples. The surface washing effect of the solvent can be further observed in cross‐sectional SEM images of planar perovskite film treated with G‐I and G‐I/TL solution (Figure S9, Supporting Information). To make the results more precise, we further comprised the devices performance only treated by pure IPA and mixture solvents without GABr. It can be found that the addition of TL in mixture solutions greatly reduced the influence of IPA on the performance parameters of solar cells (Figure S10, Supporting Information). As a result, using a single IPA solvent only increases PSCs performance barely (Figure 1c). Further, as shown in Figure 2d and Figure S11, Supporting Information, we also used XRD to reveal the structural evolution of these films and discovered all films have similar features. It can be observed in Figure 2d that the peaks around 13.98° and 28.22° correspond to the (110) and (220) planes of cubic perovskite phase (*α*‐phase), diffraction peak at 11.4° belongs to hexagonal perovskite phase (*δ*‐ phase), 12.7° belongs to PbI_2_.^[^ ^39^, ^40^ ^]^ By a careful comparison of the intensity of these XRD peaks, it is found that the perovskite films treated with G‐I solution show a much higher proportion of *δ*‐phase perovskite and the films treated with G‐I/TL solution possess the highest intensity of *α*‐phase. It means that MS‐GABr post‐treatment is helpful in grain growth and suitable phase (*α*‐phase) formation.

In Figure 2e,f, the steady‐state PL and time‐resolved PL (TRPL) spectra of three types of perovskite films are displayed. Figure 2e shows that the films treated with MS‐GABr have a greater absorbance than those treated with IPA alone, which is consistent with the EQE and short circuit current density observations. As expected, the steady‐state PL spectrum displays a greater luminescence intensity after MS‐GABr solution treatment, which is commensurate with its photovoltaic performance. Furthermore, the sample treated with G‐I/TL solution has a long carrier lifetime of 71.6 ns, as seen in Figure 2f of TRPL spectra. In contrast, the samples with and without G‐I treatment have a shorter lifetime of 46.2 and 62.1 ns, respectively. The longer carrier lifetime implies lower defect density and favorable energy alignment, demonstrating that the MS‐GABr solution treatment process can significantly improve carrier transport in perovskite films. All the above results demonstrate that MS‐GABr post‐treatment on perovskite films can improve the crystalline quality, reduce defect density, and improve the performance of final devices.

As shown in Figure 2g–i, to pinpoint the regions of perovskite affected by GABr with different solvent systems, positive ion mode time‐of‐flight secondary‐ion mass spectrometry (ToF‐SIMS) depth profiles were performed to track the distributions of the GA^+^. The ToF‐SIMS results show that the GA^+^ in the sample treated with G‐I solution has a higher intensity (Figure S12a, Supporting Information). The depth profile of samples treated with G‐I and GA‐I/TL solutions are shown in Figure S12b,c, Supporting Information. It can be found that the depth distribution of GA^+^ ions is relatively uniform in G‐I/TL treated device, which is consistent with the top‐view SEM result because the intensity variation was strongly influenced by the surface quality and the roughness of perovskite film. Moreover, we tested the contact angles when the single IPA solvent and the IPA/TL mixed solvents were dropped onto the surfaces of perovskite films at room temperature in ambient air. As shown in Figure S13, Supporting Information, the contact angle of IPA/TL mixed solvents is much smaller than that of IPA solvent on the perovskite films, indicating that IPA/TL mixed solvent has a higher wettability but lower viscosity than the pure IPA solvent. Therefore, as shown in Figure S14, Supporting Information, when the intensity of GA^+^ in different solvents is normalized to exclude the effect of viscosity, the GA‐I/TL solution has a longer diffusion length than the G‐I solution. Deeper diffusion favors the passivation of deep‐level defects near the bottom of the perovskite, resulting in less non‐radiative recombination.

**Figure 3:**
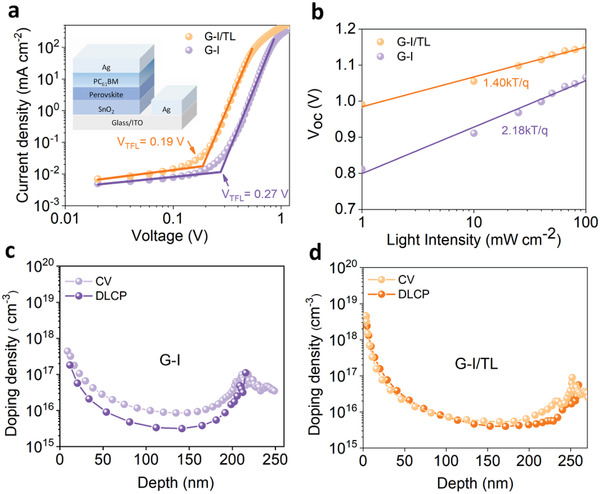
a) SCLC measurements of electron‐only devices based on G‐I/TL and G‐I solution treatment. The device structure here consists of ITO/SnO_2_/perovskite/PCBM/Ag. b) Light intensity dependence of Voc of the G‐I and G‐I/TL treated device. c,d) The apparent carrier depth profiles measured from C‐V and DLCP of the G‐I and G‐I/TL treated devices.

To clarify the carrier transport performance of perovskite treated with different types of solvents, the defect density is evaluated using the space charge limited current (SCLC) method on the structure of ITO/SnO_2_/perovskite/PCBM/Ag. The trap‐state density can be estimated by trap‐filled limit voltage (V_TFL_) through the following Equation (2)^[^ ^41^ ^]^

(2) $$N_{t}=2\varepsilon_{r}\varepsilon_{0}V_{\mathrm{TFL}}/qL^{2}$$

where V_TFL_ is the trap‐filled limit voltage, *q* is the elementary charge (*q* = 1.6×10^–19^ C), *L* is the thickness of perovskite film, *ε_0_* (*ε*_0_ = 8.854×10^–12^ F m^−1^) and *ε*_r_ (*ε*_r_ = 28.8) are the vacuum permittivity and the relative permittivity of the perovskite film. The lower trap‐state density (N_t_) in the mixture solvent treated film is shown in **Figure** **3**a and Table S2, Supporting Information, implying that the solution treatment with mixture solvent may better minimize or passivate defects in the perovskite film. Since defects are widely recognized for generating carrier traps and non‐radiative recombination centers, the FF and Voc of the mixture solvent treated PSCs with fewer defects are increased accordingly (Figure 1b).

Furthermore, we investigated the charge extraction and recombination processes in these solar devices using light‐intensity‐dependent J‐V measurements. As displayed in Figure 3b, the relationship between light intensity (I) versus V_OC_ and J_SC_ is investigated. The dependence of V_OC_ on I can be expressed by V_OC_ = ɛkTln(I)/q. It is known that when the slope ɛ deviates from (KT/q), it reflects trap‐assisted carrier recombination.^[^ ^42^ ^]^ The slope ɛ of the G‐I/TL treated device (1.40) is lower than that of the G‐I treated device (2.18), indicating that there is a less recombination loss in perovskite devices after MS‐GABr treatment process, especially in the junction region. Besides, as seen in Figure S15, Supporting Information, the dependence of *J*_SC_ on I can be defined as *J_SC_* ∝ I^*α*^ .^[^ ^43^ ^]^ Both the sample treated with G‐I and G‐I/TL solvent possess a similar *α* values of 0.994 (essentially equal to 1), signifying negligible bimolecular recombination in the devices.^[^ ^44^ ^]^ As is well known, the capacitive voltage (C‐V) response reflects free carrier, bulk, and interface defects, whereas the drive level capacitance curve (DLCP) response simply reflects free carrier and bulk defects. As a result, the presence of interface defects is frequently linked to the difference between C‐V and DLCP responses.^[^ ^45^, ^46^ ^]^ As displayed in Figure 3c,d, the C‐V curve of PSCs treated with G‐I solution deviates from DLCP curve to a certain extent, whereas the C‐V curve of PSCs treated with G‐I/TL solution overlaps with the DLCP curve, indicating that PSCs treated with a single IPA solvent still has high‐density of interface defects. However, the interface defects can be eliminated with MS‐GABr post‐treatment, which is consistent with SCLC (Figure 3a) and I‐V results (Figure 1c). Moreover, the charge transfer and recombination dynamics in the perovskite films were investigated by electrochemical impedance spectroscopy (EIS), and the associated Nyquist plots are presented in Figure S16, Supporting Information. The additive treatment notably increased the recombination resistance of the perovskite, indicating reduced charge recombination in the perovskite layer.^[^ ^47^ ^]^

**Figure 4:**
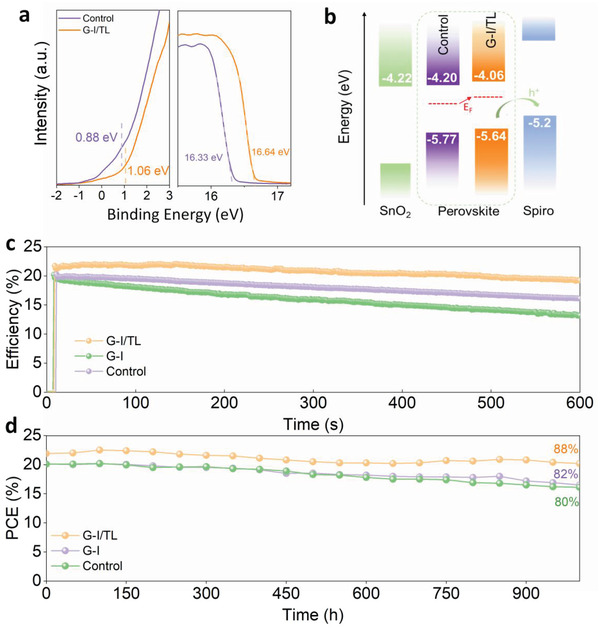
a) Secondary electron cut‐off and valence band of perovskite thin films without and with the G‐I/TL solution treatment deduced from Helium I*α* (*hν* = 21.22 eV) UPS spectra. b) Energy level diagrams of control and G‐I/TL treated films. c) The SPO (25 °C, 65% relative humidity) for the champion device without treatment, with G‐I/TL, and G‐I solution treatment. d) Long‐time stability tests of unencapsulated solar cell devices maintained in a relatively low humidity environment (about 10%).

As discussed above, the perovskite films treated with solutions of different types of solvents produce a visible difference in optical properties as well as the performance of final devices. Thus, it is necessary to determine the device parameters from the energy level perspective. The band edge positions of perovskite treated with different solvents were measured by UPS, and the detailed results are shown in **Figure** **4**a. Interestingly, the work function of the perovskite film treated by G‐I/TL solution decreases from 4.89 eV to 4.58 eV. Meanwhile, the valence band edge (E_V_) of the G‐I/TL‐treated and untreated perovskite films is 1.06 eV and 0.88 eV lower than the Fermi level (E_F_), respectively. Combined with the measurement results of bandgap (Figure S17, Supporting Information, Control 1.57eV, G‐I/TL 1.58 eV) and work function/valence band of perovskite film, the comparison diagram of energy level before and after treatment can be viewed in Figure 4b. After GABr treatment, the E_v_ shifts by about 130 meV toward the vacuum energy level, and the Fermi level rises to the vacuum energy level by about 300 meV, indicating that a more n‐type perovskite film is produced by the G‐I/TL solution treatment. The E_F_ upshift contributes to the formation of perovskite homojunction, and facilitates hole extraction from perovskite to HTL.^[^ ^48^, ^49^, ^50^ ^]^ In addition, in perovskite films, the predominant trap species leading to trap‐assisted recombination are electron traps.^[^ ^51^ ^]^ Therefore, having more n‐type perovskite film can lead to a higher percentage of occupied versus vacant traps and a slower rate of trap‐assisted recombination.^[^ ^52^ ^]^ We used Kelvin probe force microscopy (KPFM) to further assess the uniformity of the G‐I/TL treated film (Figure S18, Supporting Information) and discovered that the surface potential fluctuation is similar to that of other typical perovskite films.^[^ ^53^ ^]^

We measured the steady power output (SPO) and long‐term stability of PSCs in the atmospheric environment (25 °C, 65% relative humidity), as shown in Figure 4c, to further investigate the operation stability of PSCs after G‐I and G‐I/TL solution treatment. The devices with G‐I/TL solution treatment are found to have a clear advantage in SPO and long‐term stability tests. Even when maintained in a dry cabinet at 10% relative humidity without encapsulation, as shown in Figure 4d, the performance of G‐I/TL treated devices has retained 88% PCE after around 1000 h at room temperature. In contrast, the control group and G‐I treated devices exhibit a faster rate of PCE degradation. We further measured long‐term stability in a relatively high humidity environment (about 60%), as shown in Figure S19a, Supporting Information; unencapsulated devices cannot withstand such a high humidity environment, therefore the efficiency of G‐I/TL treated devices was reduced to 69% in only 1 week. However, the stability of the device with G‐I/TL solution treatment was still stand out in all compared samples. Additionally, the picture of the fading device has been shown in Figure S19b, Supporting Information. The instability may be due to the intrinsic properties of the perovskite material, the components of the cell, and/or the cell packing method.

**Figure 5:**
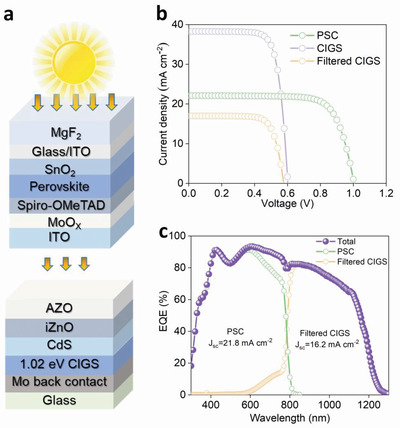
a) Schematic of the 4T perovskite/CIGS solar cell. b) J‐V curves of CIGS, filtered CIGS, and PSC. c) EQE curves of perovskite cells, CIGS filtered, and the summed.

**Figure** **5**a schematically illustrates the 4T mechanically‐stacked perovskite/CIGS tandem configuration that is used in this work. Semitransparent perovskite solar cells based on MS‐GABr solution treatment are presented with sputtered tin‐doped indium oxide (ITO) on MoO_X_ buffer as the transparent electrode. On the glass side, MgF_2_ film is used as an anti‐reflection layer to reduce reflection and hence enhance transmittance in the long‐wavelength spectral region. The best semitransparent perovskite top cell exhibits an efficiency of 18.3% from the forward scanning, with a Voc of 1.03 V, Jsc of 22.1 mA cm^–2^, and FF of 79.7%. In parallel, an optical filter of the same perovskite cell structure was fabricated, whose transmittance is shown in Figure S20, Supporting Information, and is mechanically stacked on top of our CIGS bottom cell to investigate its 4T tandem performance. The device structure of planar CIGS is shown in the bottom of Figure 5a and the stand‐alone CIGS solar cell has a PCE of 17.5% under the standard AM1.5G spectrum. As the CIGS under tandem configurations only receive the light filtered by the top PSCs, they effectively perform under a much lower injection level compared to standard AM1.5G conditions, with the main efficiency drop due to the decrease of Jsc from 37.7 to 16.2 mA cm^–2^ (Figure S21, Supporting Information). The effective add‐on efficiency from the CIGS under the PSCs is 7.2%, as present in **Table** **1**. The corresponding I‐V test results are shown in Figure 5b. The EQE curves of perovskite cells, CIGS filtered and their total is presented in Figure 5c. The performance of n‐i‐p structure 4T perovskite/CIGS tandem cells compared with previous is shown in **Table** **2**. By summing the efficiency of the filtered CIGS device and that of the perovskite top cell, a 4T tandem efficiency of 25.5% has been obtained, which is comparable to the highest efficiency of 25.9% p‐i‐n structure 4T perovskite/CIGS tandem solar cell.^[^ ^16^ ^]^

**Table 1:** Photovoltaic parameter of different types of solar cells and the overall efficiency of 4T perovskite/CIGS tandem solar device

Devices	PCE (%)	Voc (V)	FF (%)	Jsc (mA cm^–2^)
Semi‐transparent perovskite top cell	18.3	1.03	79.7	22.1
CIGS bottom cell	17.5	0.61	76.4	37.7
Filtered CIGS	7.2	0.58	76.2	16.2
4T tandem cell	25.5			

**Table 2:** Comparison of performance between the present and previous 4T perovskite/CIGS tandem cells

PSCs structure	Top PSC	CIGS	Filtered CIGS	Total	Year ref.
Glass/ITO/TiO_x_/Perovskite/Spiro‐OMeTAD/ MoO_X_ /Au/ MoO_X_	11.5%	12.4%	4.0%	15.5%	2015^[^ ^54^ ^]^
LiF/Glass/FTO/TiO_2_/Perovskite/Spiro‐OMeTAD/AgNW	12.7%	17.0%	5.9%	18.6%	2015^[^ ^14^ ^]^
MgF_2_/Glass/FTO/mp‐TiO_2_/Perovskite/Spiro‐OMeTAD/ MoO_X_ /ZnO:Al/ MgF_2_	12.1%	18.4%	7.4%	19.5%	2015^[^ ^55^ ^]^
Glass/FTO/TiO_2_/Perovskite/Spiro‐OMeTAD/ITO	12.0%	15.7%	5.8%	17.8%	2017^[^ ^56^ ^]^
Glass/FTO/TiO_2_/Perovskite/Spiro‐OMeTAD/Ag/ITO	16.0%	12.3%	4.7%	20.7%	2017^[^ ^57^ ^]^
MgF_2_/Glass/ITO/TiO_2_/Perovskite/Spiro‐OMeTAD/MoO_X_/IZO/ MgF_2_	18.1%	16.5%	5.8%	23.9%	2018^[^ ^15^ ^]^
MgF_2_/Glass/ITO/NP‐SnO_2_/Perovskite/Spiro‐OMeTAD/MoO_X_/ITO	17.5%	21.2%	7.5%	25.0%	2020^[^ ^58^ ^]^
MgF_2_/Glass/ITO/PTAA/Perovskite/ C_60_/SnOx/Zn:SnOx(ZTO)/IZO (p‐i‐n structure)	17.1%	20.7%	8.8%	25.9%	2019^[^ ^16^ ^]^
MgF_2_/Glass/ITO/SnO_2_/Perovskite/MS‐GABr/Spiro‐OMeTAD/MoO_X_/ITO	18.3%	17.5%	7.2%	25.5%	This work

## 3 Conclusion

In summary, we have demonstrated that solution post‐treatment with mixture solvents could effectively improve the quality of perovskite films as well as the performance of final devices. It is found that G‐I/TL solution treatment can effectively suppress the formation of the perovskite *δ*‐phase, improves the crystallinity of perovskite films, increases carrier lifetime, passivates defects and thus minimizes non‐radiative recombination rate, and prevents carrier quenching at the perovskite interface. Photovoltaic devices fabricated using the G‐I/TL solution treatment strategy have demonstrated a champion PCE of 22.7%, together with significant improvements in Voc, Jsc, and FF. The success of the MS‐GABr strategy presents us a simple and versatile approach to improve the performance of perovskite solar cells. Replacing the metallic back electrode by ITO on MoOx buffer, a semitransparent perovskite solar cell has been fabricated based on the above treatment with the best efficiency reaching 18.3%. Coupling with a 17.5% efficiency CIGS bottom cell, we have also realized a 4T perovskite/CIGS tandem solar cell with 25.5% efficiency. Impressively, this is the most efficient 4T perovskite/CIGS tandem solar cell of the n‐i‐p structure ever reported.

## 4 Experimental Section

### Materials

DMF (anhydrous, 99.8%), DMSO (anhydrous, ≥99.9%), isopropanol (IPA, anhydrous, 99.5%), chlorobenzene (CB, anhydrous, 99.8%), Toluene (TL, anhydrous, 99.8%), ethyl acetate (EA, anhydrous, 99.8%), and MoO_x_ were purchased from Sigma‐Aldrich. SnO_2_ colloidal solution (15 wt% in water) was purchased from Alfa Aesar. Lead (II) iodine (PbI_2_, ≥99.9%), Formamidinium iodide (FAI, ≥99.5%), methylammonium bromide (MABr, ≥99.5%), Spiro‐OMeTAD (≥99.5%), 4‐tert‐butyl pyridine (tBP), ≥96%), lithium bis(trifluoromethanesulfonyl) imide (Li‐TFSI, ≥99%), and tris(2‐(1h‐pyrazol‐1‐yl)‐4‐tert‐butylpyridine)‐cobalt(III)tris(bis(trifluoromethylsulfonyl)imide) (FK 209 Co(III) TFSI, ≥99%) were purchased from Xi'an Polymer Light Technology in China. Methylammonium chloride (MACl, ≥99.5%), cesium iodide (CsI, ≥99.99%) was purchased from Advanced Election Technology CO, Ltd. in China. The ITO target (In_2_O_3_/SnO_2_ = 90:10 wt%) was purchased from Huizhou Tianyi Rare Materials Co., Ltd in China.

### Solution Preparation

The solution was prepared in an inert atmosphere inside a nitrogen glove box. The Cs_0.05_MA_0.10_FA_0.85_Pb(I_0.97_Br_0.03_)_3_ perovskite precursor solution was prepared by dissolving 19.8 mg CsI, 442.2 mg FAI, 74.22 mg PbI_2_, 74.22 mg MACl, and 74.22 mg MABr in 1 mL DMF and DMSO mixture solvent (3:1 v/v), and stirred overnight. The perovskite solution was filtered before solution‐casting. The 2 mg mL^−1^ GABr was dissolved either in pure IPA, or mixed solution with TL, CB, and EA. The Spiro‐OMeTAD solution was prepared by dissolving 72.3 mg Spiro‐OMeTAD, 18 µL Li‐TFSI solution (520 mg of Li‐TSFI in 1 mL of ACN), 18 µL FK 209 Co (III) TFSI solution (360 mg of FK 209 Co (III) TFSI in 1 mL of ACN) and 28 µL tBP in 1 mL chlorobenzene. SnO_2_ colloid particles were diluted with deionized H_2_O in the ratio of 1:2 before use.

### Device Fabrication

1) For the opaque perovskite solar cells, ITO glass substrates (1.5 cm × 1.5 cm) were sequentially cleaned by deionized water, acetone, and IPA for 30 min, respectively. Then, the ITO glasses were dried by a nitrogen gun and treated with UV‐ozone at 100 °C for 30 min. SnO_2_ based electron transport layer was spin‐coated on the pre‐cleaned ITO glasses at 3000 rpm for 30 s, followed by annealing at 150 °C for 20 min in air. This SnO_2_ coating was repeated once under the same conditions. After depositing the electron transport layer, the samples were treated with UV‐ozone for 30 min before transferring into the glove box. The perovskite layer was then deposited under an inert atmosphere inside the nitrogen glove box. A consecutive two‐step spin‐coating procedure was performed by 1000 rpm for 10 s followed by 5000 rpm for 30 s. At about 8 s before the end of the spin‐coating step, 100 µL of EA was dropped onto the sample. The samples were then put onto a hotplate for 10 min at 130 °C. Afterward, 50 µL of GABr solution was dropped on the annealed perovskite film during a spin‐coating procedure at 5000 rpm for 60 s. Subsequently, the hole transport layer was deposited on the top of the perovskite film by spin coating the Spiro‐OMeTAD solution at 3000 rpm for 40 s. Finally, 80 nm of Au was thermally evaporated as the electrode under a high vacuum using an e‐beam thermal evaporator.
2) For the semitransparent device, about 10 nm of MoO_x_ was evaporated on top of Spiro‐OMeTAD at a rate of 0.1 A s^−1^ at 4 × 10^−4^ mbar pressure. The rear transparent contact was then fabricated by RF sputtering 100 nm of ITO on the MoO_x_ buffer. The sputtering was performed with 40 W RF power and 0.15 Pa Ar pressure for 40 min. To complete the semitransparent device, Ag fingers were then scattered around the semitransparent perovskite solar cells and the total active area of the semitransparent cells is 0.07 cm^2^. MgF_2_ layer was deposited at the rate of 1.0 Å s^–1^ on the glass side as anti‐reflection coating with a thickness of 90 nm. It shall be noted that using, for example, the paraffin oil as an optical coupler would result in slightly more effective suppression of multiple reflections between the top and bottom cells in a 4T tandem configuration. For the measurements of the bottom cells in a 4T configuration, semitransparent perovskite filters with the same structure and optical properties as that of the semitransparent perovskite solar cells were prepared with a larger substrate size (1.5 cm^2^).

### Characterization

The surface and cross‐sectional morphologies of the perovskite were obtained with a field‐emission scanning electron microscope (Zeiss SIGMA). The crystal structure and phase of the perovskite were characterized using XRD (Rigaku Smartlab‐3 kW). The absorption spectra were measured using a UV–visible spectrophotometer (Agilent Carry5000). The photoluminescence (PL) and time‐resolved photoluminescence (TRPL) measurements were performed with a DeltaFlex fluorescence spectrometer (HORIBA) installed with an excitation source of 485 nm picosecond pulsed diode laser. The element distribution of the solar cells was obtained by secondary ion mass spectroscopy (TOF‐SIMS 5–100, ION‐TOF GmbH). A Bi liquid metal ion source (LMIS) was employed for mass data acquisition. Mass data was acquired using the Bi^3+^ cluster ion. The depth profiles and high‐resolution imaging were detected utilizing the Bi^3+^ primary ion beam, and analyzed over a 150×150 µm^2^ area with a 128:128 primary beam raster. A Cs ion source at 10 keV was employed as the sputter/etch tool. The Cs^+^ sputtered/etched area was 300 × 300 µm^2^ and the area analyzed by the LMIS was at the center of the sputtered/etched crater formed by the rastering Cs^+^ beam. The I‐V curves were recorded using a Keithley 2400 Source Meter under standard AM 1.5 G illumination using a solar simulator (Enli Technology Co., Ltd). The devices were measured both in reverse scan (1.2 V → ‐0.2 V, step: 0.02 V) and forward scan (‐0.2 V → 1.2 V, step: 0.02 V). The external quantum efficiency (EQE) spectra were recorded using a QE/IPCE system (Enli Technology Co. Ltd). The space‐charge limited current (SCLC) method was used to measure the dark I‐V curve on phenyl‐c61‐butyric‐acid‐methyl‐ester (PCBM) coated Glass‐ITO/SnO_2_/Perovskite sample to derive the defect concentration.

## Conflict of Interest

The authors declare no conflict of interest.

## Supporting information

Supporting Information

## Data Availability

The data that support the findings of this study are available in the supplementary material of this article.
