# Supplementary material for: Mixed Solvents Assisted Post‐Treatment Enables High‐Efficiency Single‐Junction Perovskite and 4T Perovskite/CIGS Tandem Solar Cells
Source: Adv Sci (Weinh). 2022 Jun 8;9(23):2201768. doi: 10.1002/advs.202201768 (PMC9376828; doi:10.1002/advs.202201768)
Supplement: Supplementary file 1 — Supporting Information [file ADVS-9-2201768-s001.pdf]

Supporting Information

Mixed solvents assisted post-treatment enables high-efficiency single-junction perovskite and 4T perovskite/CIGS tandem solar cells

Liting Tang, Xiaomin Wang, Xinxing Liu, Junjun Zhang, Shaoying Wang, Yuqi Zhao, Junbo Gong, Jianmin Li*, and Xudong Xiao*[a]***

Liting Tang, Xinxing Liu, Junjun Zhang, Shaoying Wang, Yuqi Zhao, Junbo Gong, Jianmin Li, Xudong Xiao
Key Laboratory of Artificial Micro- and Nano-structures of Ministry of Education, and School of Physics and Technology, Wuhan University, Wuhan 430072, China.
E-mail: gongjunbo@whu.edu.cn, ljmphy@whu.edu.cn, xdxiao@whu.edu.cn

Xiaomin Wang
Center for Biomedical Optics and Photonics (CBOP) & College of Physics and Optoelectronics Engineering, Key Laboratory of Optoelectronic Devices and Systems, Shenzhen University, Shenzhen, 518060, P. R. China

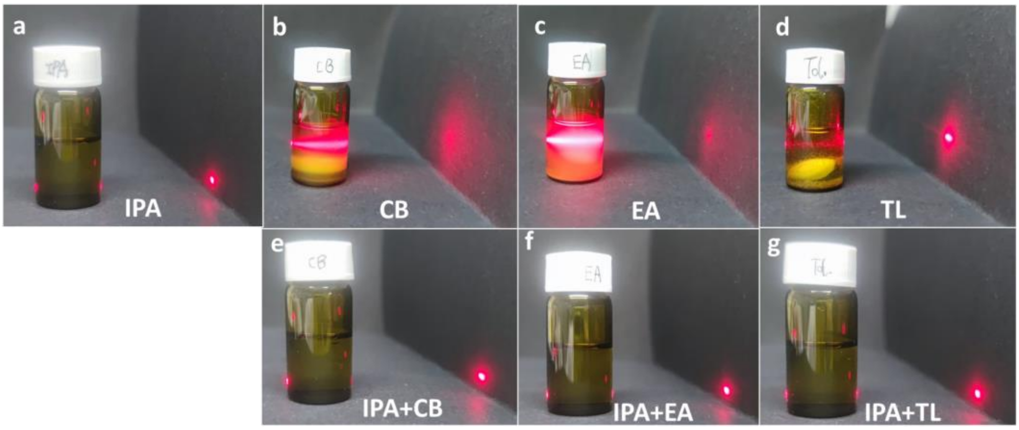

Figure S1 The dissolution of GABr in different solvents. (a) 2 mg/ml GABr dissolved in IPA. (b) 2 mg/ml GABr dissolved in CB. (c) 2 mg/ml GABr dissolved in EA. (d) 2 mg/ml GABr dissolved in TL. (e) 2 mg/ml GABr dissolved in 1:1 mixture of IPA and CB. (f) 2 mg/ml GABr dissolved in 1:1 mixture of IPA and EA. (g) 2 mg/ml GABr dissolved in 1:1 mixture of IPA and TL.

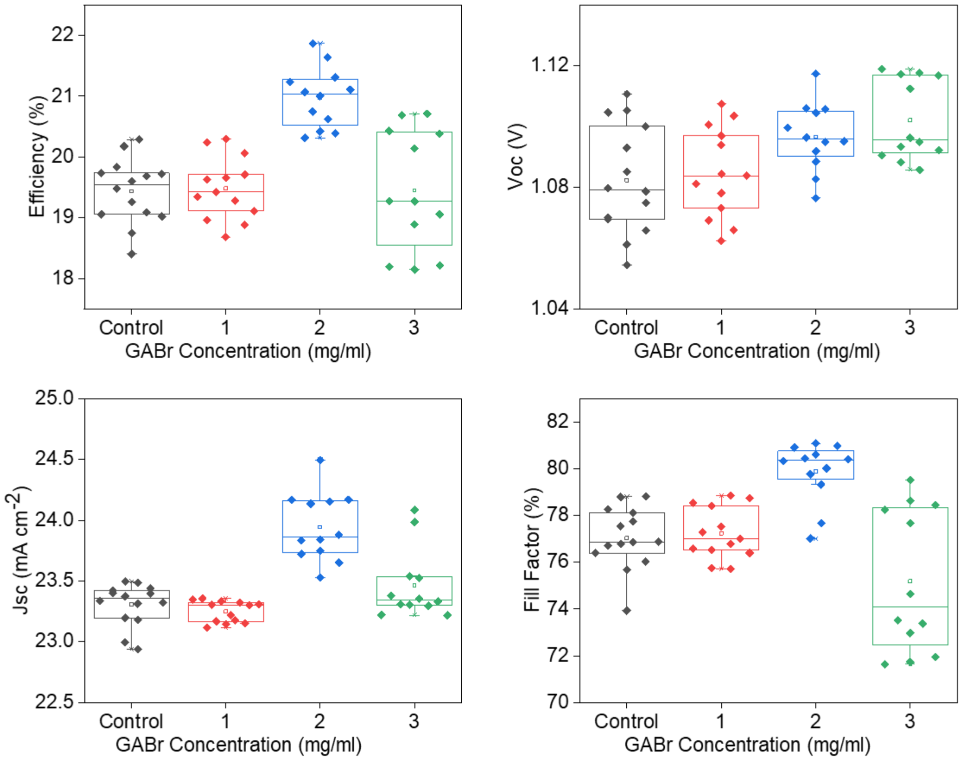

Figure S2 Statistical photovoltaic parameters (PCE, Voc, Jsc, and FF) of devices treated with various concentrations of GABr (IPA: TL=1:1).

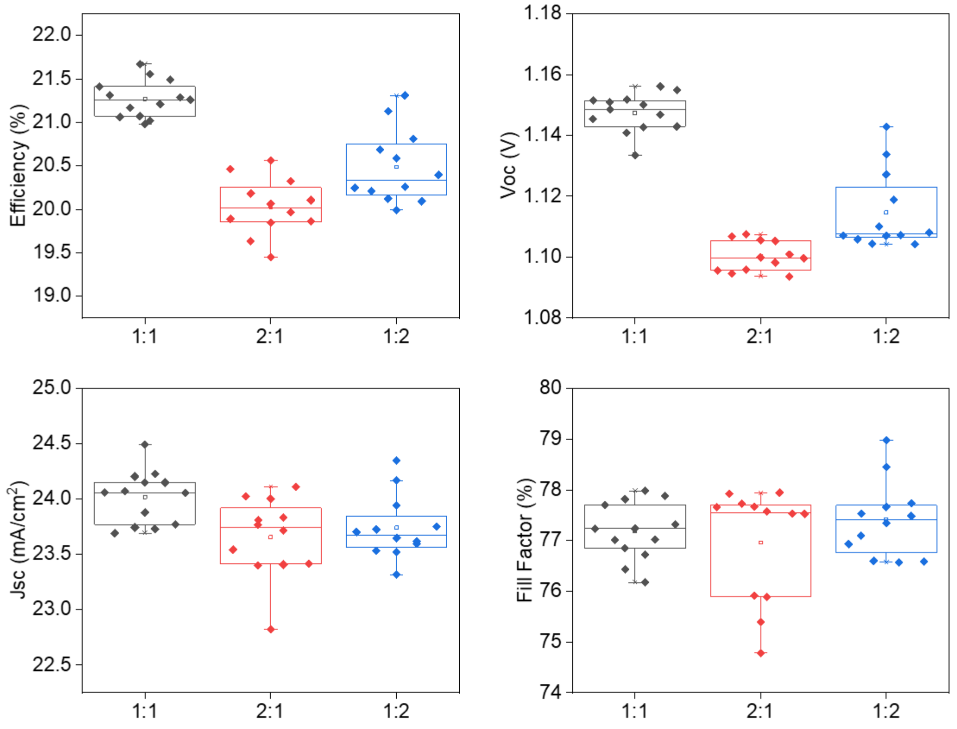

Figure S3 Photovoltaic parameters (PCE, Voc, Jsc and FF) of solar cells based on the ratios of IPA:TL.

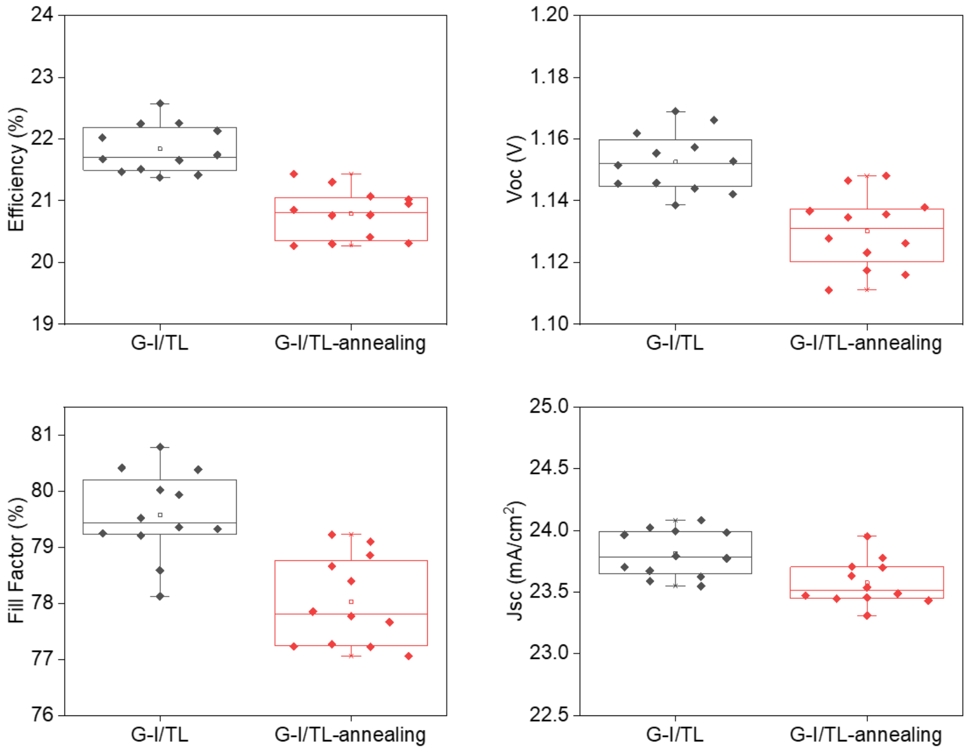

Figure S4 Photovoltaic parameters (PCE, Voc, Jsc and FF) of G-I/TL treated solar cells with and without annealing at 100°C for 5 min.

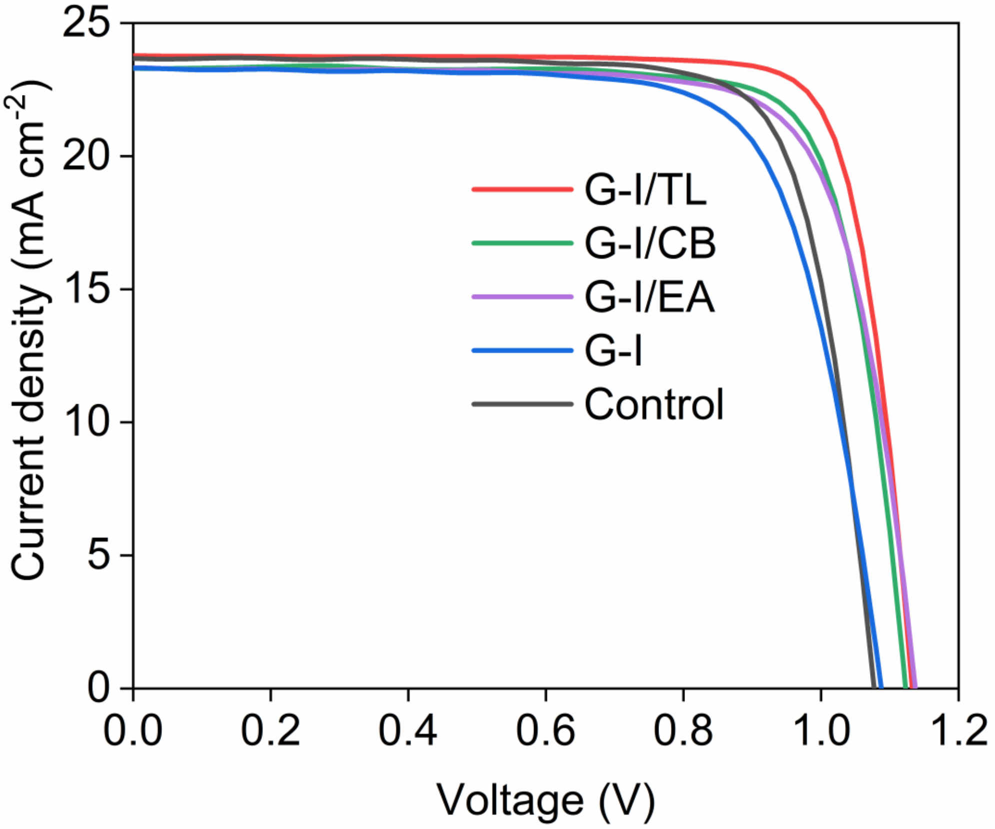

Figure S5 J-V curves of solar cells with and without GABr treatment in different solvents.

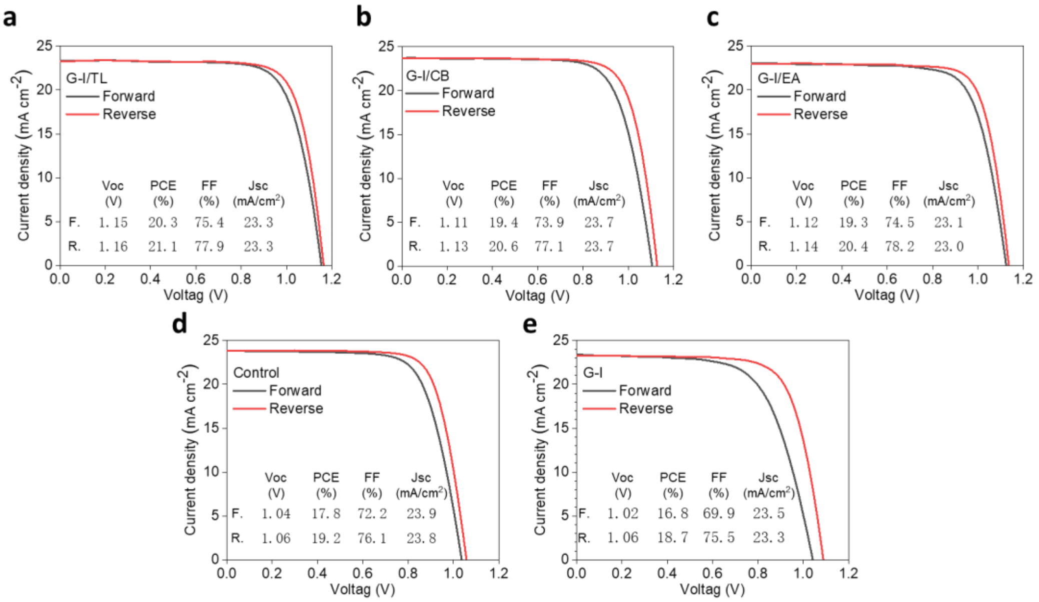

Figure S6 Forward and reverse scan J-V curves of solar cells with and without GABr treatment in the different solvents.

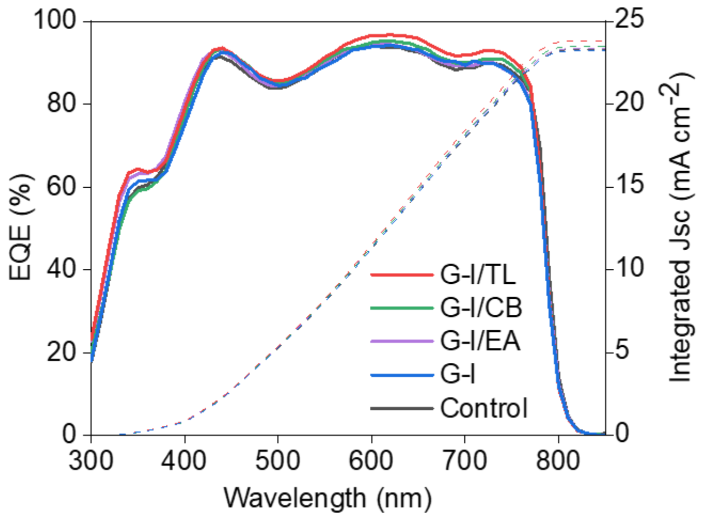

Figure S7 EQE curves of solar cells with and without GABr treatment in different solvents.

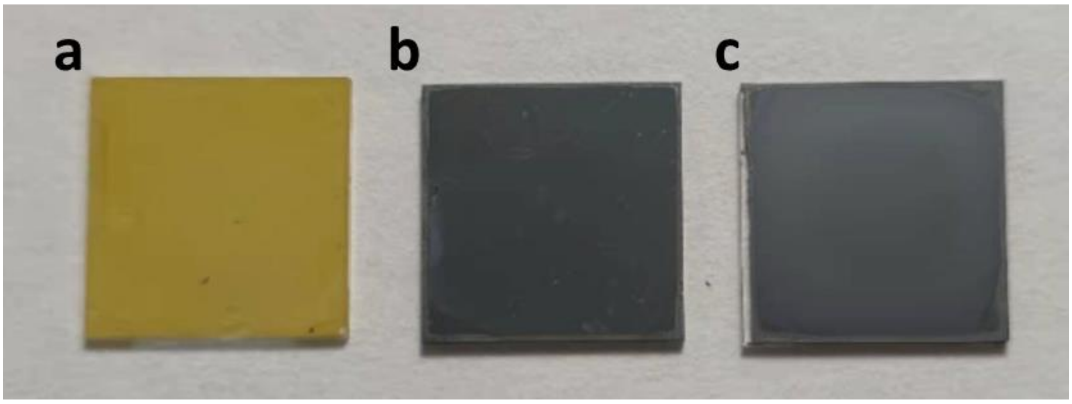

Figure S8 Photograph of perovskite thin films exposed to pure IPA solvent (a), IPA/TL mixed solvent (b), and pure TL solvent (c) for 6 hours.

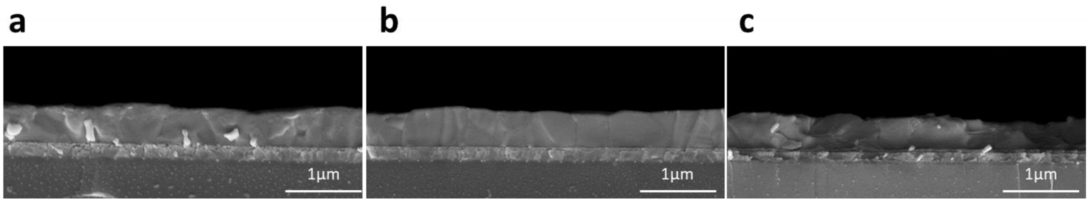

Figure S9 Cross-sectional SEM images of planar perovskite film without treatment (a), with G-I/TL (b), and G-I (c) solution treatment.

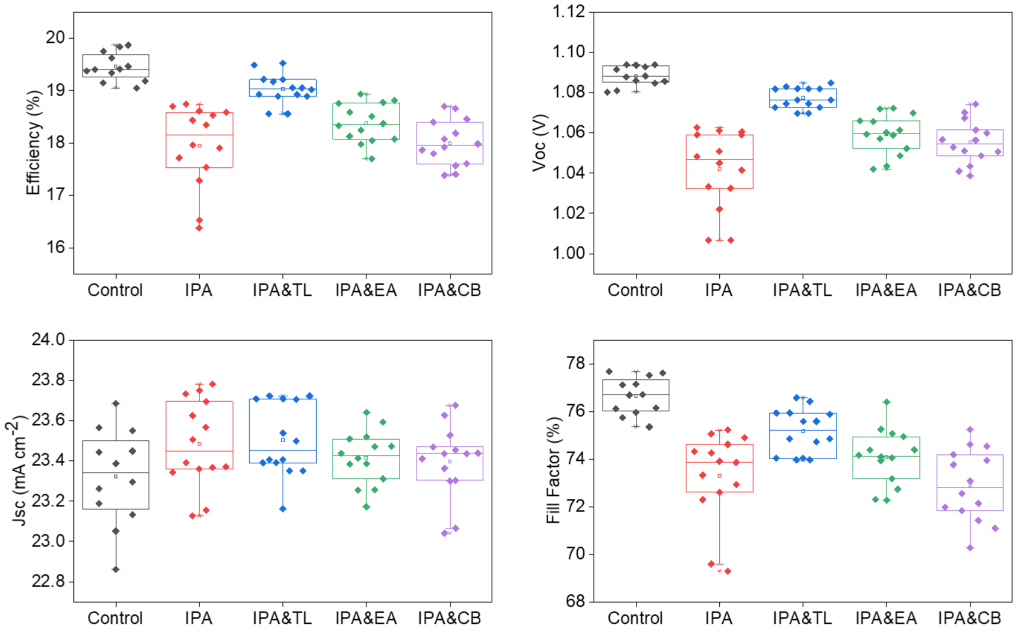

Figure S10 Performance parameters of solar cells treated with pure IPA and mixture solvents without GABr. The control devices are also listed.

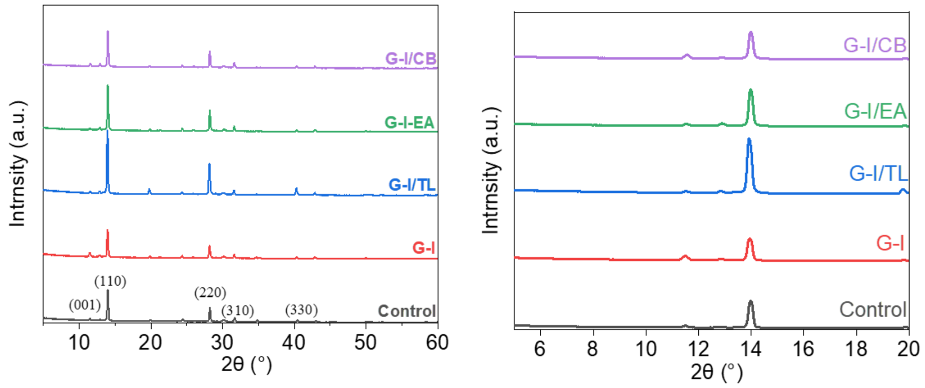

Figure S11 XRD patterns of perovskite films with and without GABr treatment in different solvents.

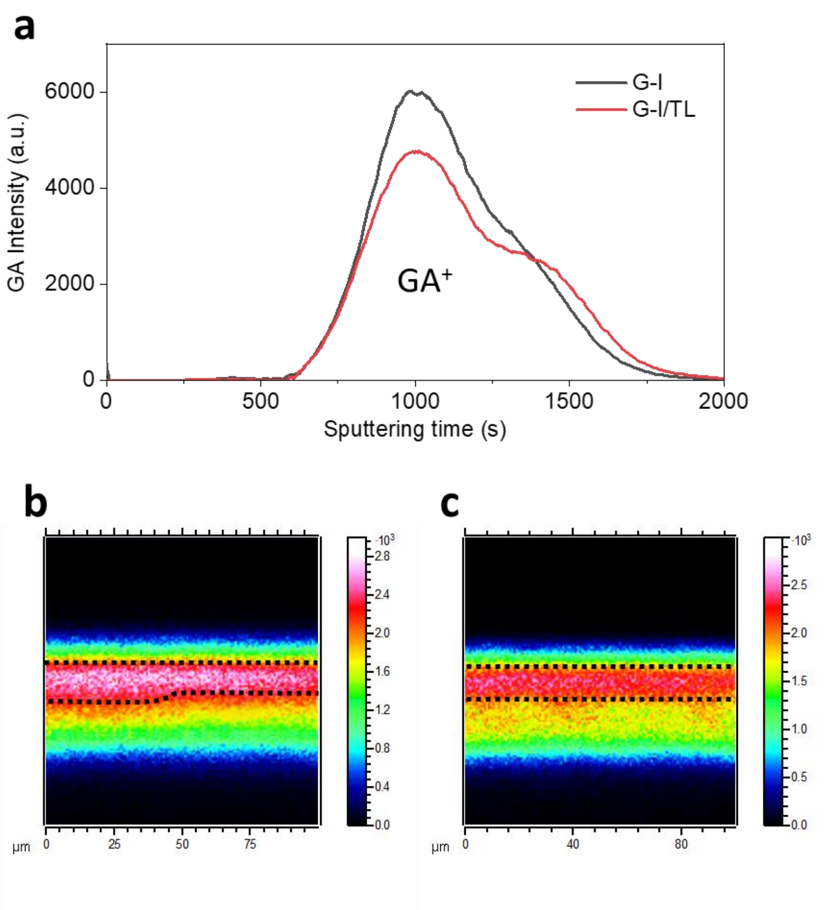

Figure S12 (a) SIMS signals intensity from GA$^+$ of devices treated with G-I and G-I/TL solution. (b-c) Depth profile of samples treated with G-I and GA-I/TL solution.

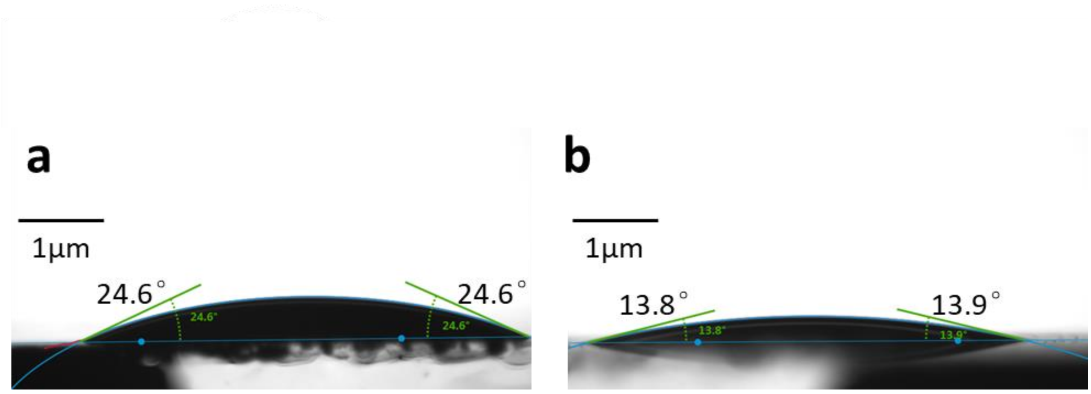

Figure S13 The contact angle tests of pure IPA solvent (a) and IPA/TL mixed solvents (b) on the perovskite films.

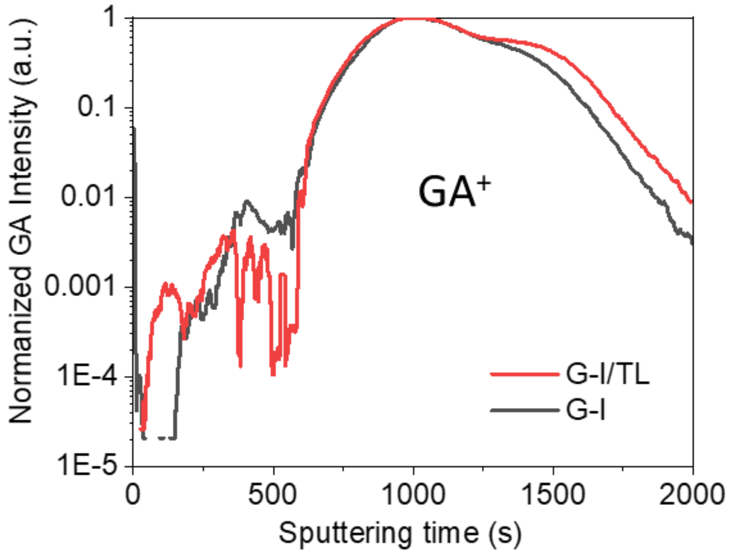

Figure S14 Normalized the intensity of GA^+ of devices treated with G-I and G-I/TL solution.

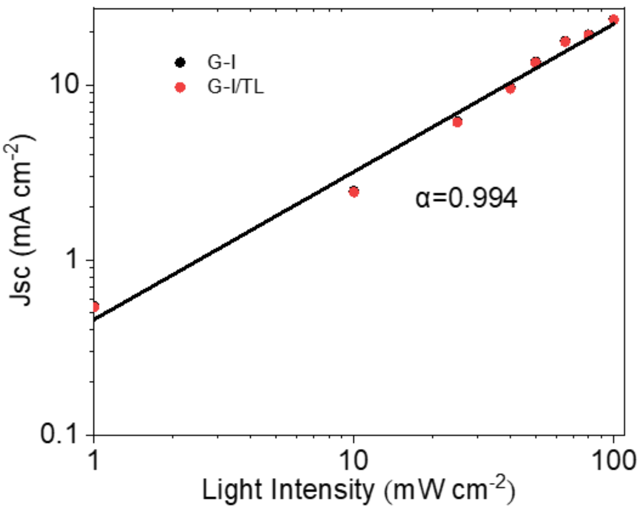

Figure S15 Light intensity dependence of Jsc of the G-I and G-I/TL treated device.

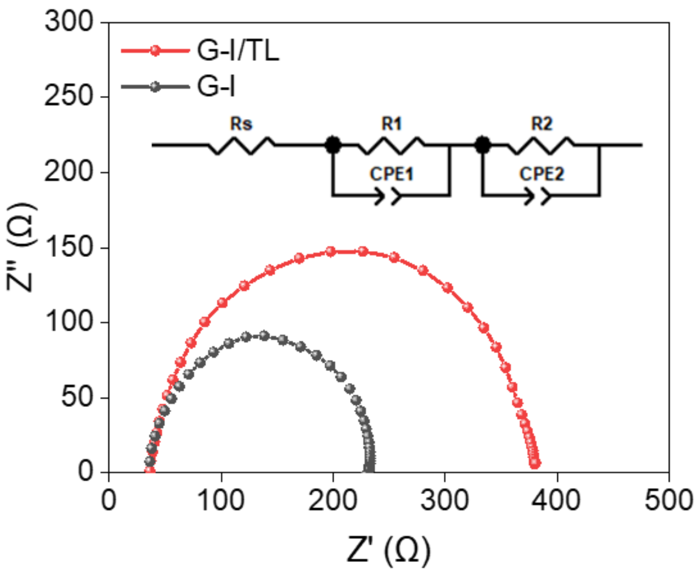

Figure S16 Nyquist plots of the EIS measurements of the G-I and G-I/TL treated devices, and (inset) a circuit diagram of the equivalent circuit model.

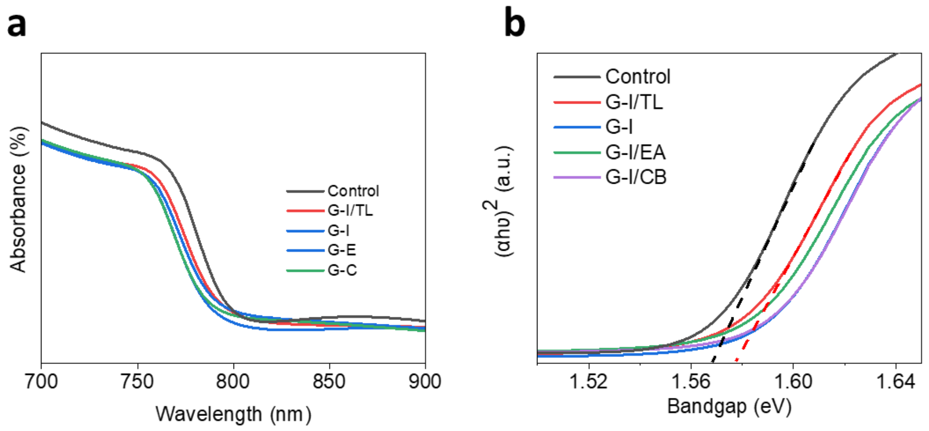

Figure S17 (a) UV-vis absorption spectra and (b) Tauc plots of perovskite films with and without GABr treatment in different solvents.

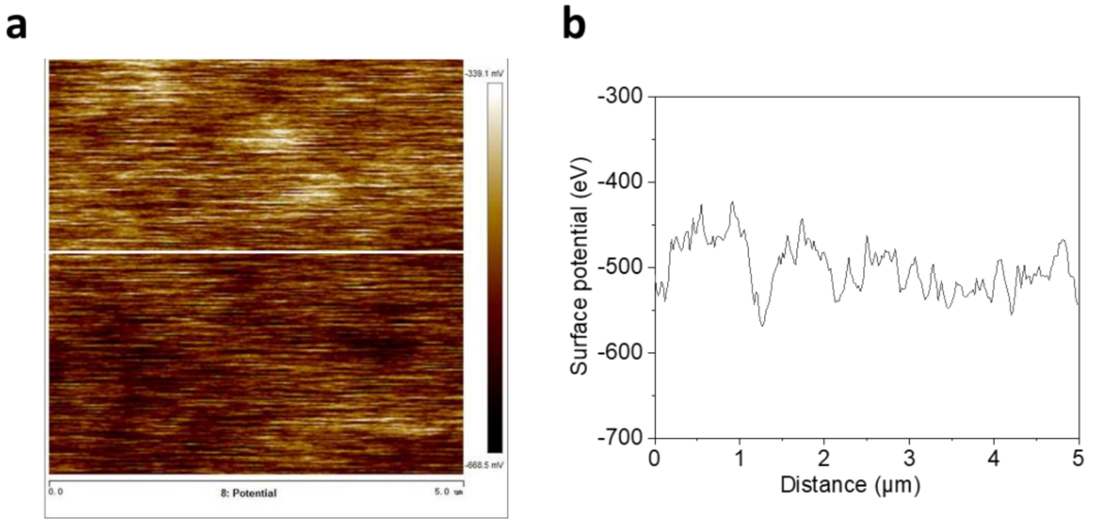

Figure S18 (a) 2D mapping of Kelvin probe force microscopy for the G-I/TL film. (b) The variation of surface potential along the white line marked in the 2D mapping.

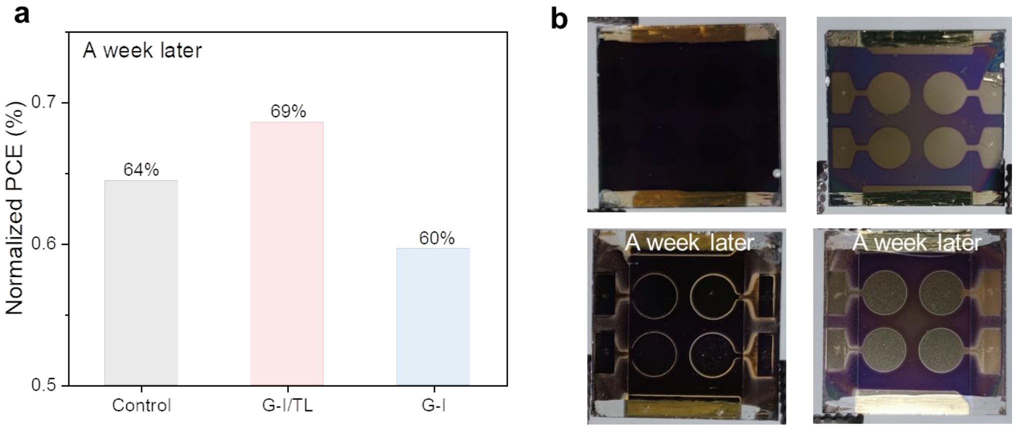

Figure S19 (a) Long-time stability tests of unencapsulated solar cell device maintained in a relatively high humidity environment (about 60%) . (b) The picture of the fading device.

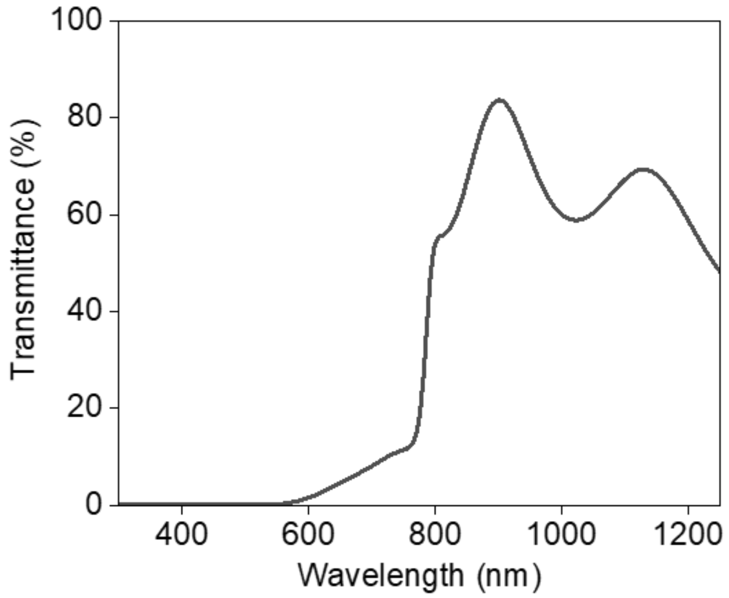

Figure S20 Transmittance of the semitransparent perovskite filter used for 4T tandem measurements.

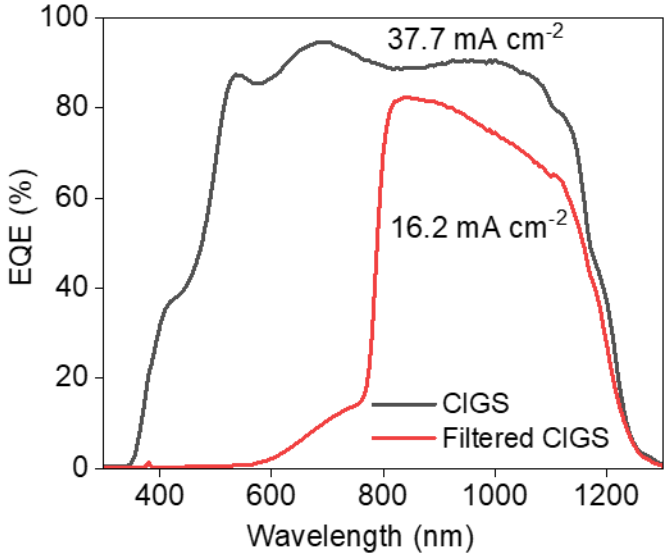

Figure S21 EQE spectra of CIGS and the filtered CIGS devices.

Table S1 Hysteresis of solar cells with and without GABr treatment in different solvents

**Samples**	**Control**	**G-I**	**G-I/TL**	**G-I/CB**	**G-I/EA**
**Hysteresis**	7.2%	10.1%	3.8%	5.8%	5.4%

Table S2 Electrical property parameters of solar cells with and without GABr treatment in different solvents

**Samples**	**A**	**V_{TFL} (V)**	**N_t (cm^{-3})**
**G-I**	2.18	0.27	2.39×10^{15}
**G-I/TL**	1.40	0.22	1.66×10^{15}
